# Coupled rhizosphere application of cyanobacteria-bamboo acid hydrolysis extract and cyanobacterial biochar enhances soil health and crop quality

**DOI:** 10.1186/s40643-026-01016-5

**Published:** 2026-02-11

**Authors:** Huichang Bian, Yuzhi Li, Yibiao Zhang, Yao Shen, Jiahou Hao, Shuo Wang, Ji Li

**Affiliations:** 1 Jiangsu Key Laboratory of Anaerobic Biotechnology, School of Environment and Ecology, Jiangnan University, 1800 Lihu Avenue, Wuxi, 214122 Jiangsu China; 2 Department of Civil Engineering and Architecture, Nanyang Normal University, Nanyang, 473061 China; 3 Jiangsu College of Water Treatment Technology and Material Collaborative Innovation Center, Suzhou, 215009 China

**Keywords:** Cyanobacteria, Bamboo powder, Plant stimulants, Soil improvement, Soybean quality, Risk assessment

## Abstract

Driven by waste resource utilization and carbon neutrality imperatives, this study synthesized cyanobacterial growth elicitor (CGE) and cyanobacterial-bamboo growth elicitor (CBGE) via acid-hydrothermal hydrolysis. The coupling potential of cyanobacterial biochar (CB) for improving rhizosphere soil and crop quality was investigated through four pot trial treatments: (1) CK (control), (2) BR (rhizospheric CB), (3) LBR (rhizospheric CB with CGE), (4) LZBR (rhizospheric CB with CBGE). Integration of phenotypic analyses, microbiome profiling, functional gene predictions, and risk assessment elucidated biostimulant mechanisms. Compared to CK, all treatments elevated soil nutrient levels. BR exhibited superior nitrogen enrichment (15 ± 3 g/kg), while LBR and LZBR—particularly LZBR—enhanced phosphorus/potassium bioavailability and maximized soil organic carbon (SOC). LZBR treatment markedly increased Chryseobacterium abundance (an organic matter-decomposing genus). Functional verification confirmed enhanced C-N-P cycling activity, minimized environmental nutrient leakage, and improved plant nutrient assimilation. Specifically, LZBR increased soybean grain protein content by 37.0 g/kg and plant nitrogen accumulation by 5.4 g/kg compared to CK, and risk assessments indicated no detectable ecotoxicological effects. Consequently, the coupled application of CGE and CBGE derived from cyanobacteria and bamboo powder simultaneously improves soil quality and crop performance. This approach establishes a novel waste valorization pathway, suitable for partial replacement of chemical fertilizers and carbon emission reduction.

**Graphical abstract:**

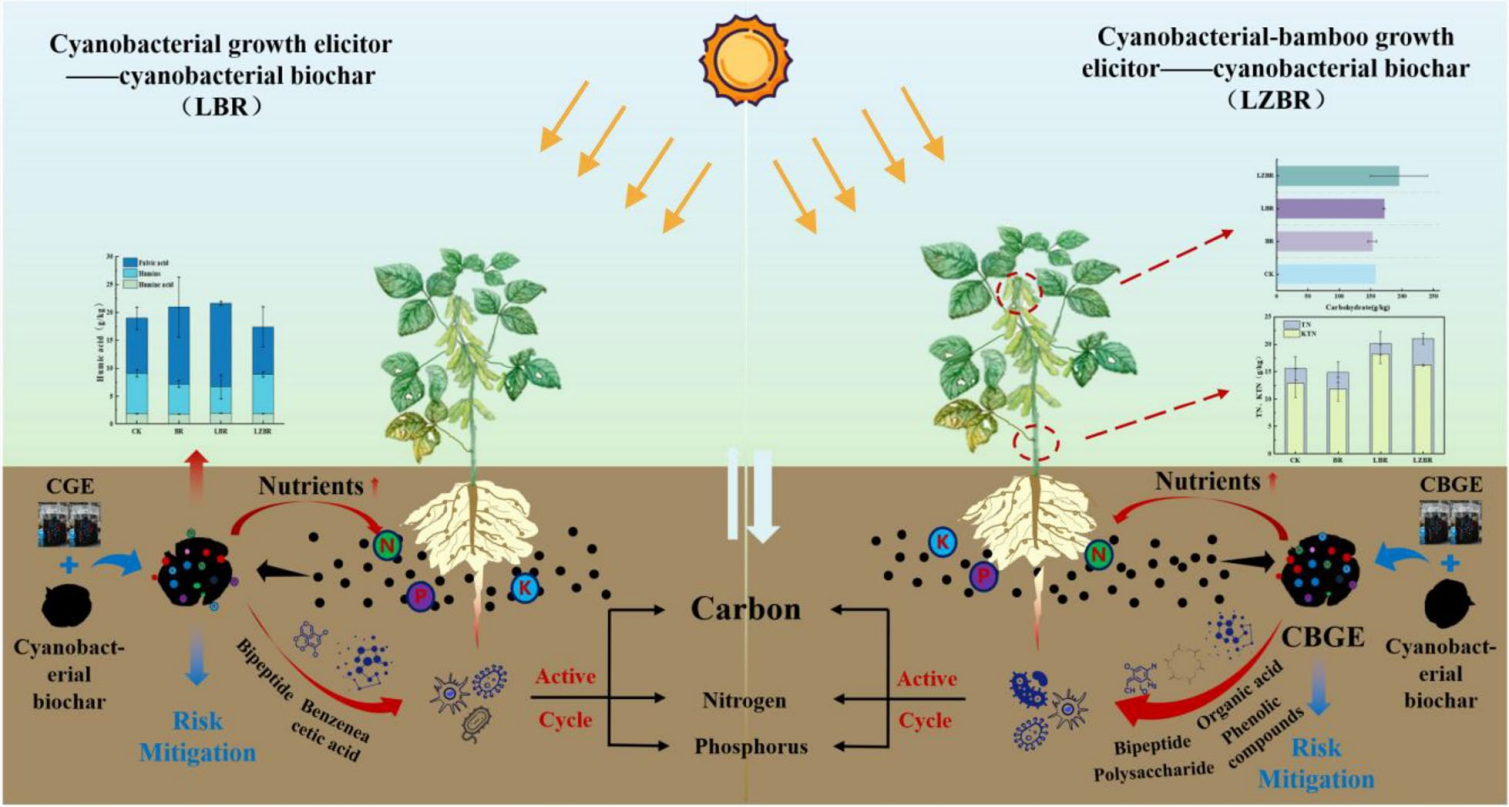

**Supplementary Information:**

The online version contains supplementary material available at 10.1186/s40643-026-01016-5.

## Highlights

CB preferentially adsorbs polysaccharides, dipeptides, and organic acids in CGE and CBGE.

LBR and LZBR more effectively boost soil P and K levels with no environmental health risk.

CGE and CBGE enhance soil CN P cycling processes.

CGE and CBGE significantly improve soybean nutritional quality.

CBGE demonstrates superior soil quality amelioration and crop quality enhancement over CGE.

**Supplementary Information:**

The online version contains supplementary material available at 10.1186/s40643-026-01016-5.

## Introduction

Climate change critically constrains agricultural sustainability (Sun et al. 2022). In 2023, global agricultural activities generated 4 billion tons of CO_2_e emissions, accounting for 11% of anthropogenic greenhouse gases (Zhou et al. 2025), indicating substantial mitigation potential within this sector (Li et al. 2025). Although chemical fertilizers boost crop yields, their excessive application elevates carbon emissions (Parasar and Agarwala 2025), compromises soil health and crop quality, and disturbs ecosystems (Liu et al. 2019; Wen et al. 2024; Quitzow et al. 2025). Consequently, the development of fertilizer alternatives characterized by multiple benefits—capable of reducing carbon emissions, improving soils, and enhancing crop quality—has emerged as a prominent focus in contemporary agricultural research (Han et al. 2025; You et al. 2022; Chen et al. 2024a; Ayaz et al. 2025).

Research on converting waste into nitrogen-phosphorus-rich liquid organic fertilizers integrated with plant biostimulants has attracted considerable research interest. Barbara et al. (Scaglia et al. 2015) identified substantial quantities of plant biostimulants and their precursors in digestate from anaerobic digestion of pig manure and agricultural waste. Through alkaline pyrolysis applied to dissolved organic matter in anaerobic digestion sludge, Du et al. (Du et al. 2023) transformed it into high-quality liquid fertilizer abundant in amino acids and humic substances, which markedly enhanced rice growth. Hao et al. (Hao et al. 2024a, 2023, 2024b) developed SS-NB—a sludge-derived micronutrient biostimulant with dual functions as fertilizer and biostimulant—via alkaline thermal hydrolysis. This biostimulant demonstrated effectiveness in soil remediation, increased yield and quality of pak choi, reduced heavy metal accumulation in crops, and provided economic advantages through partial substitution of chemical fertilizers.

Cyanobacterial bloom management and sludge disposal present dual environmental challenges in China’s Taihu Lake basin (Huisman et al. 2018; Li et al. 2022; Chen et al. 2024b). Official reports indicate 1.72 million tons of cyanobacteria were removed annually in 2024. Unlike eukaryotic algae, these cyanobacteria contain significant protein, lipid, carbohydrate, and polyunsaturated fatty acid reserves (Nunes et al. 2024)—largely untapped biomass resources. While established thermochemical valorization routes encompass biofuel generation (Kim et al. 2022) and biochar synthesis (Chen et al. 2024c), agricultural applications remain limited. Parallelly, bamboo powder dominates lignocellulosic residues from southern China’s bamboo processing industry. Its cellulose-rich matrices (42–50%) are embedded in lignin–hemicellulose complexes (Wang et al. 2025; Silva et al. 2024), with secondary components including pigments, pectin, and inorganic compounds. Current resource recovery prioritizes biochar production and aldehydic compound extraction (Zhang et al. 2023; Li et al. 2023; Wang et al. 2024a). Consequently, synergistic conversion of both waste streams into multifunctional plant biostimulants offers simultaneous resource reclamation, eutrophication mitigation, and net carbon emission reduction.

Extending documented soil amelioration by acid-thermal hydrolyzed cyanobacterial growth elicitor (CGE) and cyanobacterial-bamboo growth elicitor (CBGE), pot experiments coupled each elicitor separately with biochar. Within adsorption-facilitated systems, three key parameters were quantified: (1) soil physicochemical properties, (2) rhizospheric microbial community composition, and (3) crop quality metrics. Resulting analyses revealed rhizosphere-specific mechanistic insights for soil-crop system enhancement alongside application risk assessment. This integrated methodology thereby constructs a theoretical foundation for implementing biochar-coupled CGE/CBGE systems in sustainable agriculture, ensuring operational safety while advancing carbon mitigation goals.

## Materials and methods

### Material preparation

Cyanobacterial biomass was sourced from a dewatering facility in Wuxi, China, with bamboo powder (200-mesh particle size) procured from Ningguo Zijie Wood Industry Co., Ltd. (China). Detailed preparation methodologies for CGE and CBGE appear in supplementary material Text S1. Cyanobacterial biochar was produced by Jiangsu Jinshan Environmental Technology Co., Ltd. (China) via pyrolysis of dried Taihu Lake cyanobacteria at 450 °C under a N_2_ atmosphere. The resulting biochar had a specific surface area of 11.2 m^2^/g, an average pore diameter of 13.5 nm, a pore volume of 0.033 cm^3^/g, and a pH of 5.44 (determined at a solid-to-liquid ratio of 1:20). It also contained surface functional groups (e.g., carbonyl, hydroxyl, aromatic structures) conducive to adsorption and reactivity. Experimental soil was collected from the plough layer (0–20 cm depth) of a typical Black Soil region in Heilongjiang Province, Northeast China. Soybean seeds (Glycine max cv. Dongsheng 79) were provided by the Mudanjiang Branch of the Heilongjiang Academy of Agricultural Sciences.

### Biochar adsorption experiment

Biochar-CGE/CBGE composites (100:1 mass ratio) were prepared. Mixtures were agitated for 24 h at 25 °C and 200 rpm using an orbital shaker (Thermo Scientific MaxQ 4000, USA). Post-adsorption supernatants underwent 0.45 μm membrane filtration for polycyclic aromatic hydrocarbons (PAHs) and nutrient quantification. BET analysis (Micromeritics ASAP 2460, USA) quantified biochar pore characteristics—including size distribution and specific surface area—following 12 h degassing at 150 °C (Poddar et al. 2024).

### Nutrient composition analysis

#### Plant nutrients

Total Kjeldahl nitrogen (TKN), total phosphorus (TP), phosphate (PO_4_^3−^), and total potassium (TK) were determined to evaluate plant-nutrient abundance in CGE and CBGE. All analytical procedures strictly adhered to the Chinese Industry Standard NY 525-2012 (Organic Fertilizers).

#### Phytohormones and allelopathic substances

Phytohormones and allelochemicals in CGE and CBGE were analyzed following the methods provide by Hao et al. (Hao et al. 2024c). Specifically, 500 μL supernatant underwent mixing with 20 μL of 0.3 mg/mL myristic acid internal standard in 0.8 mL methanol/water (3:1, v/v). The protocol proceeded with: sequential vortex mixing (5 min), ultrasonic extraction (60 Hz, 10 min), 10 min incubation at 4 °C, and centrifugation at 13,500 × g (10 min). The resulting supernatant (1000 μL) was vacuum-dried at 45 °C, followed by addition of 80 μL methoxyamine hydrochloride (15 mg/mL), vortexing (2 min), and 90 min incubation at 37 °C. Subsequent derivatization required adding 80 μL N-methyl-N-(trimethylsilyl)trifluoroacetamide and 20 μL n-hexane, vortexing (2 min), and heating at 70 °C for 60 min. Final detection employed gas chromatography-time-of-flight mass spectrometry (GC-TOFMS; Pegasus HRT + 4D, LECO Corporation, USA), using internal standard peak area ratios for semi-quantification.

### Polycyclic aromatic hydrocarbons (PAHs) analysis

PAH extraction utilized hexane/acetone (1:1, v/v). Extracts underwent purification via C18 solid-phase extraction cartridges (500 mg/6 mL, Thermo Scientific), preconditioned sequentially with 5 mL methanol and 5 mL deionized water. Concentration proceeded under gentle N_2_ stream, followed by PTFE membrane filtration (0.22 μm) prior to analysis. GC–MS/MS analysis (Trace 1600-TSQ 9610, Thermo Fisher Scientific, USA) employed a TG-5SilMS capillary column (30 m × 0.25 mm × 0.25 μm), with chromatographic separation at 1.0 mL/min helium flow.

### Experimental design

Four treatments were established, each with two independent pot replicates: (a) CK (control); (b) BR (rhizospheric cyanobacterial biochar, CB); (c) LBR (rhizospheric CB + CGE); (d) LZBR (rhizospheric CB + CBGE). Each pot was filled with 5 kg of homogenized black soil (sieved to < 2 mm). For treatments BR, LBR, and LZBR, the respective amendments (pristine CB or CB pre-adsorbed with CGE/CBGE) were homogeneously blended into the lower 2.5 kg of soil at an application rate of 0.1% (w/w). Two soybean seeds were sown per pot. All pots were irrigated daily to maintain soil moisture at field capacity. No additional fertilizers were applied during the 90-day cultivation period. Rhizospheric and bulk soil samples were collected before planting and after harvest according to the Chinese industry standard HJ/T 166-2004.

### Soil physicochemical property analysis

Analysis of rhizospheric/non-rhizospheric soil parameters—including total nitrogen (TN), phosphorus (TP), potassium (TK), ammonium (NH_4_^+^-N), nitrate (NO_3_^−^-N), soil organic carbon (SOC), humin, humic acid, and fulvic acid—followed standardized protocols from *Methods in Agricultural Chemical Analysis: A Practical Handbook* (Faithfull 2002). Heavy metal quantification complied with China National Standard HJ 1315-2023 (*Total 19 Metal Elements in Soils/Sediments *via* ICP-MS*). Soil health risk assessment employed three indices: geo-accumulation index (I_geo_) (Muller 1969), potential ecological risk index (PI) (Hakanson 1980), and carcinogenic risk index (CR) (Cui et al. 2022), with computational methods detailed in supplementary materials Text S1, background values for soil heavy metals are presented in Table S1.

### Soybean indices determination

Post-harvest soybean tissues underwent homogenization and milling prior to total nitrogen quantification with an elemental analyzer (Vario MACRO cube, Elementar). Grain protein determination employed a Kjeldahl nitrogen analyzer (Kjeltec 8400, FOSS).

### Soil microbial community analysis

Soil microbial diversity was analyzed via metagenomic sequencing (MajorBio Co., Ltd., Shanghai, China) targeting the V3–V4 region (primers 338F/806R) (Han et al. 2024). For both rhizosphere and non‑rhizosphere soils, analyses were conducted with three replicate samples: two independent pot‑level biological replicates and one pooled quality‑control replicate comprising equal amounts of the two biological samples.

### Statistical analysis

To ensure reproducibility, a duplicated experimental design was implemented. Analyses were performed in triplicate, and the reported data are presented as means, with values controlled within 2–3 significant figures to reflect measurement precision. Specifically, in the presentation of data as mean ± standard deviation (SD), the mean was rounded to the decimal place corresponding to the first significant digit of its respective SD. For measured variables (e.g., soil nutrient concentrations, plant growth parameters, and yield components), statistical differences among the treatments were evaluated by one-way analysis of variance (ANOVA). Where a significant effect was identified, Tukey’s honestly significant difference (HSD) post hoc test was applied for multiple comparisons. Data processing, graphical construction, and microbiome analyses (including functional gene prediction) were performed using Microsoft Excel 2023, Origin 2021 (OriginLab, USA), and the MajorBio Cloud Platform (https://cloud.majorbio.com), respectively.

## Results and discussion

### Characteristics of CGE and CBGE

#### Basic nutrients

Continuous nutrient influx maintains soil ecological balance. Table 1 details key nutrients in CGE and CBGE. Crucially, CBGE contained 88% higher total nutrients (5.8 × 10^4^ mg/kg) than CGE (3.1 × 10^4^ mg/kg). As a fundamental macronutrient for legumes, nitrogen governs protein and chlorophyll synthesis (Hao et al. 2024c), making soil nitrogen status and precision fertilization recognized as vital for crop yield and soil fertility. Total nitrogen was measured at 2.18 × 10^3^ mg/kg in CBGE versus 1.22 × 10^3^ mg/kg in CGE (79% higher), with parallel advantages observed in TKN and NH_4_^+^-N fractions. Elevated TP, TK, and organic matter (OM) in CBGE derived from cellulose (40–50% dry weight) and mineral-rich ash in bamboo powder. Soil carbon storage can be enhanced by such persistent organic inputs (Chang et al. 2024).

**Table 1:** Compositional comparison between CGE and CBGE

Sample	pH	TKN (mg/kg)	NH_4_^+^-N (mg/kg)	NO_3_^−^-N (mg/kg)	TP (mg/kg)	TN (mg/kg)	TK (mg/kg)	OM (mg/kg)
CGE	5.4	774	470	302	136	1.22 × 10^3^	411	2.76 × 10^4^
CBGE	5.6	1.06 × 10^3^	1.10 × 10^3^	38.0	228	2.18 × 10^3^	765	5.27 × 10^4^

#### Allelochemicals and phytohormones

Phenolic compounds, alkaloids, and organic acids were quantified in CGE and CBGE (Table 2). Total allelochemical content measured 63 mg/kg in CGE versus 364 mg/kg in CBGE. Alkaloid concentrations decreased in both elicitors due to thermal decomposition under acidic high-temperature conditions (Wieczorek et al. 2015). Lower phenolic levels in CGE showed negligible plant growth inhibition, whereas higher phenolic content in CBGE stemmed primarily from lignin pyrolysis-derived monophenols during bamboo processing (Wang et al. 2018). Significantly higher organic acid concentrations were detected in CBGE than CGE, potentially enhancing mineral activation, alleviating cadmium/aluminum toxicity, improving microbial efficiency, and promoting heavy metal immobilization (Shi et al. 2024).

**Table 2:** Quantitative analysis of phytohormones and allelochemicals in CGE and CBGE

Phytohormone	CGE (mg/kg)	CBGE (mg/kg)	Allelochemical	CGE (mg/kg)	CBGE (mg/kg)
Polysaccharide	1.78 × 10^3^	6.76 × 10^3^	Phenolic compounds	4.4	134
Bipeptide	256	235			
2(5H)-Furanone	29.6	111	Alkaloid	1.3	< LOD
Pyrroles	11.3	77.7			
Benzeneacetic acid	102	89.4	Organic acid	57.6	230
Other-furans	4.8	153			
Total (mg/kg)	2.19 × 10^3^	7.43 × 10^3^	Total (mg/kg)	63.3	364

Comparative analysis revealed elevated phytohormone content in CBGE (7.43 × 10^3^ mg/kg) relative to CGE (2.19 × 10^3^ mg/kg), alongside greater polysaccharide abundance dominated by cellobiose, glucose, and xylose. While CGE polysaccharides potentially stimulate phytohormone secretion and stress tolerance via auxin analogs, pyrroles, and furans, the superior concentrations of growth regulators and biostimulants in CBGE indicate broader agronomic applicability (Liu et al. 2025; Krishna et al. 2022).

### Biochar nutrient adsorption characteristics

**Fig. 1:**
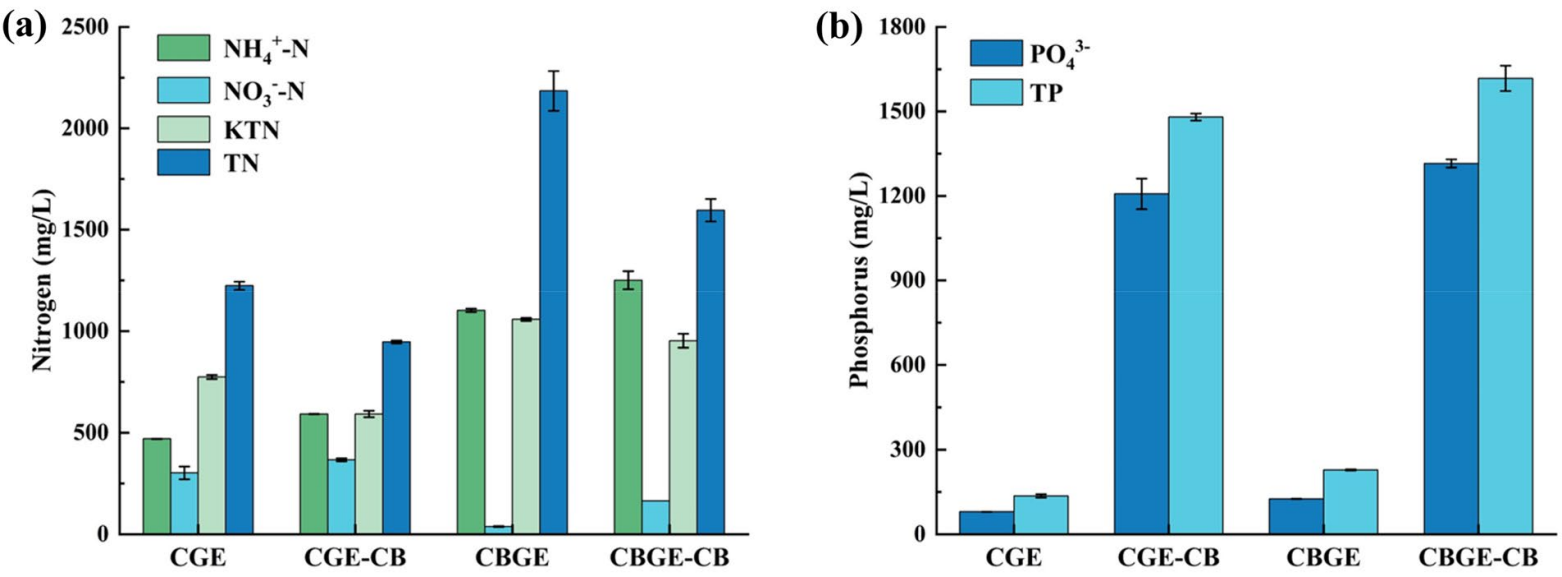
Nutrient variations in cyanobacterial growth elicitor (CGE, raw solution) and cyanobacterial-bamboo growth elicitor (CBGE, raw solution) before and after cyanobacterial biochar adsorption (-CB, post-adsorption supernatant). **a** Biochar-adsorbed nitrogen; **b** Biochar-adsorbed phosphorus

Figure 1a demonstrates effective adsorption of TKN by cyanobacterial biochar from CGE and CBGE solutions, achieving 24% and 10% TKN reduction respectively. This adsorption advantage under near-neutral to slightly acidic conditions (Hao et al. 2024c; Wang et al. 2024b) correlates with the positively charged surface of biochar that provides additional binding sites. Lower TKN adsorption efficiency was observed in CBGE than CGE, likely due to higher polysaccharide content in CBGE. The complex molecular structure of these polysaccharides occupied active sites of biochar, hindering adsorption of nitrogen-containing organic compounds. BET analysis post-CBGE treatment confirmed decreased average pore size (Table S2), indicating pore filling by polysaccharides that reduced adsorption performance. Furthermore, biochar exhibited minimal adsorption of NH_4_^+^-N and NO_3_^−^-N with concurrent inorganic nitrogen loss. Consequently, superior adsorption of organic components suggests that the primary influence of biochar on soybean growth arises from differential retention of organic fractions.

Figure 1b illustrates changes in phosphorus nutrients before and after adsorption. The biochar demonstrated limited adsorption capacity for TP and PO_4_^3−^, failing to retain these nutrients while releasing substantial phosphate. This desorption behavior aligns with ionic nitrogen adsorption patterns (Hao et al. 2024c).

### Dynamic changes in soil nutrients

#### Soil nutrient variations

The initial soil pH was 7.34. Following the experimental treatments, acidic CGE and CBGE applications decreased soil pH via H^+^ ion introduction. Compared to the initial pH (7.34), all treatments led to a reduction in soil pH. While the BR treatment (cyanobacterial biochar only) maintained a comparable pH (6.69) to the CK control (6.70), LBR and LZBR treatments exhibited significant reductions to 6.52 and 6.65, respectively. These pH alterations influence nutrient and heavy metal mobility and speciation.

**Fig. 2:**
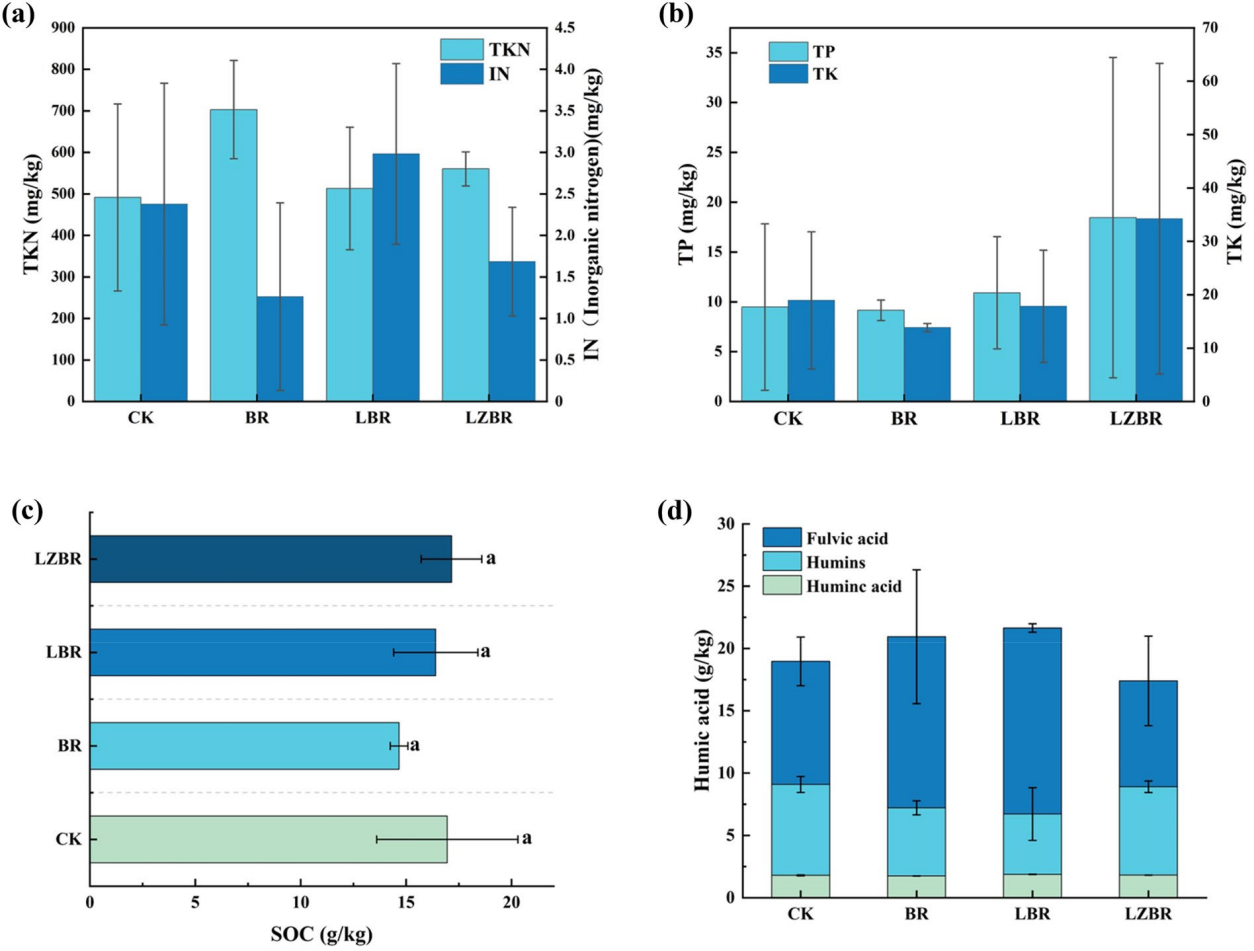
Variations in soil nutrient indicators. **a** Nitrogen content; **b** TP and TK content; **c** SOC; **d** Humic substances

Figure 2a presents nitrogen nutrition responses to the combined amendments in rhizosphere soil. Relative to the control (CK: 490 ± 230 mg/kg), all treatments showed numerically higher TKN concentrations. The largest numerical increase was observed in the BR treatment (approximately + 210 mg/kg), while LBR and LZBR showed more moderate increases (approximately + 20 and + 70 mg/kg, respectively). This attributed to nitrogen-enriched composition in cyanobacterial biochar and associated microorganisms. Gradual release of adsorbed nitrogenous organic compounds sustained elevated TKN concentrations relative to CK despite partial TN loss during CGE/CBGE adsorption.

Figure 2b illustrates TP dynamics in rhizosphere soil. BR maintained concentrations comparable to CK, while LBR and LZBR exhibited increases of 1.4 mg/kg and 8.9 mg/kg respectively. This enhancement originated from adsorption and gradual release of phosphorus derived from hydrolysis of organic matter and phosphorus-containing compounds in CGE and CBGE. The superior TP elevation in LZBR compared to LBR correlates with high-organic-matter bamboo powder in CBGE, with its hydrolytic products (nucleic acids, phospholipids) effectively augmented soil TP. Furthermore, hormonal components stimulated microbial activity, accelerating phosphorus mineralization and mobilization.

Analysis of Fig. 2b indicated reduced TK in BR and LBR rhizosphere soils relative to CK. Cyanobacterial biochar diminished soil TK via potassium ion adsorption, with further reduction arising from biochar significant potassium adsorption capacity—which disturbed K-P equilibrium and induced phosphate dissolution—plus potassium consumption during organic matter decomposition. Conversely, elevated TK content in CBGE increased LZBR soil TK by 15.3 mg/kg, enhancing post-application potassium release.

Consequently, LBR and LZBR demonstrated superior phosphorus and potassium enhancement, particularly the exceptional potassium efficacy of LZBR, while BR excelled in nitrogen improvement. These findings establish that targeted fertilizer applications optimize soil fertility and advance sustainable agriculture.

#### Soil organic carbon

SOC critically influences soil fertility, plant development, and structural stability (Xu and Tsang 2024). While cyanobacterial biochar reduced soluble SOC in BR treatments by 2.3 g/kg (Fig. 2c) through adsorption-mediated soil modifications, in LBR systems a 0.6 g/kg SOC decrease occurred via three pathways: biochar adsorption of CGE-derived organics, microbial decomposition, and additional SOC adsorption. Conversely, LZBR exhibited 0.2 g/kg SOC accumulation after biochar pre-adsorption of CBGE organics, where microbial processing generated novel organic compounds and pH reduction promoted carbon stabilization. Consequently, CBGE application enhances SOC retention with significant carbon sequestration and emission mitigation potential.

#### Humic substances

Humic substances critically regulate soil carbon cycling, plant development, pH homeostasis, fertility, and structural stability. As shown in Fig. 2d, cyanobacterial biochar increased the total humic components from 19 ± 3 g/kg in the CK to 22 ± 6 g/kg in the BR treatment, indicating a promotion of humification. The concentration in LBR (21.7 ± 0.6 g/kg) was numerically similar to that in BR. In contrast, LZBR exhibited a lower level (17 ± 4 g/kg), suggesting that the presence of CGE in LBR might have supported synthesis relative to LZBR. primarily resulting from CBGE-induced inhibition of humus formation. The high cellulose content of bamboo powder and phytochemical-mediated microbial suppression substantially inhibit humic substance synthesis (Ma et al. 2024).

Fulvic acid declined from 7.3 ± 0.6 g/kg (CK) to 5.5 ± 0.6 g/kg (BR) and 5 ± 2 g/kg (LBR), mainly due to altered soil chemistry by cyanobacterial biochar and organic inputs. These changes reduced fulvic acid stability and solubility while inducing substantial decomposition. Conversely, LZBR showed a marginal reduction to 7.1 ± 0.5 g/kg, where soluble organics in CBGE bamboo powder were selectively converted into low-molecular-weight fulvic acid (Ma et al. 2024), thereby compensating for partial consumption.

Simultaneously, humin increased from 10 ± 2 g/kg (CK) to 14 ± 5 g/kg (BR) through microbially-driven transformation of cyanobacterial biochar-derived organics. Biochar porosity further facilitates humin stabilization and accumulation. Specifically in LBR, humin rose to 14.9 ± 0.3 g/kg, suggesting that CGE conditions promote stable humin formation, particularly via redox-mediated modulation. However, LZBR humin decreased to 9 ± 4 g/kg, likely reflecting preferential synthesis of simple fulvic acid over complex humin from bamboo powder, while allelopathic compounds in CBGE induce soil instability, further impeding humin stabilization. Humic acid exhibited minimal changes.

### Responses of rhizosphere microbial communities

#### Microbial diversity

**Fig. 3:**
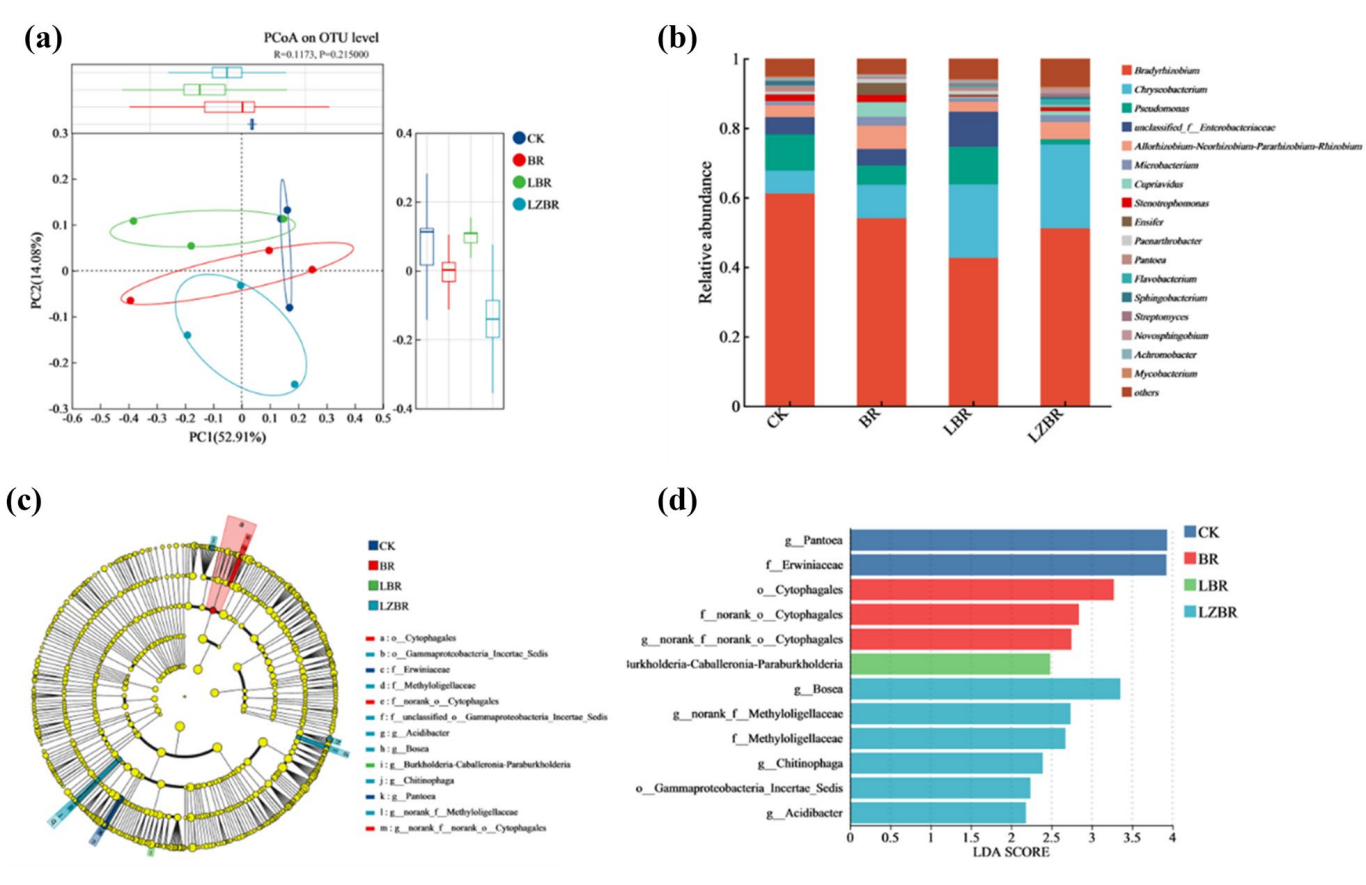
Rhizosphere microbial community responses. **a** PCoA significance; **b** Genus-level composition; **c** LEfSe differential features; **d** LDA discriminant features

Analysis of 12 rhizosphere soil samples yielded 581,532 quality-filtered sequences, which clustered into 1,419 OTUs. Alpha diversity indices (ACE, Chao, Simpson, Shannon; Table S3) indicated significantly higher microbial diversity in LBR compared to CK, whereas BR and LZBR showed reduced diversity. These findings suggest that CB, CGE, and CBGE collectively modulate rhizosphere microbial alpha diversity. Principal coordinate analysis (PCoA; Fig. 3a) separated bacterial communities, with PC1 (52.91%) and PC2 (14.08%) explaining 67.0% of total variance. The ordination revealed distinct clustering patterns per treatment.

#### Microbial community analysis

Significant restructuring of microbial communities occurred at the phylum level under BR, LBR, and LZBR treatments (Fig. S1), predominantly involving the dominant phyla Actinobacteria, Bacteroidetes, and Proteobacteria. Proteobacteria abundance decreased consistently across treatments, whereas the dominant phyla Bacteroidetes and Actinobacteria increased. These compositional shifts strongly correlated with modified soil humic/fulvic acid content, driven by biogeochemical changes from cyanobacterial biochar and adsorbates.

*Bradyrhizobium* and *Chryseobacterium* dominated genera composition (Fig. 3b). *Bradyrhizobium* facilitates legume symbiotic nitrogen fixation, enhancing plant growth and soil nutrients (Siqueira et al. 2020; Wan et al. 2024). Conversely, *Chryseobacterium* mediates organic matter decomposition, promoting plant development and stress tolerance (Tatsumi et al. 2021). Although *Bradyrhizobium* decreased from 61.17% in CK, it remained predominant, directly elevating soil nitrogen via fixation. In LBR, *Chryseobacterium* increased by 14.56% due to released CGE-derived organics and biostimulants, inducing microbial competition that reduced other taxa. Concurrently, *unclassified_f__Enterobacteriaceae* (acetate producers) increased under acidic conditions (Yin et al. 2025).

LZBR showed 17.51% higher *Chryseobacterium* abundance than LBR, primarily from CBGE-derived substrates that stimulated proliferation. Nutrient competition combined with CBGE phenolics selectively reduced other microbes. Post-incorporation, organic matter underwent decomposition by *Chryseobacterium* consortia, transforming into stable carbon forms retained in soil. This process enhances rhizosphere carbon cycling and plant assimilation, demonstrating significant carbon sequestration potential.

#### Species differentiation analysis

LEfSe analysis (LDA > 2.0) resolved treatment-specific divergence in rhizosphere microbiomes (Fig. 3c–d), detecting 11 differentially abundant taxa. BR exhibited significant enrichment of Cytophagales (order)—known carbon-cycle mediators with soil remediation potential (Tatsumi et al. 2021). LBR showed dominance of Burkholderia-Caballeronia-Paraburkholderia, wherein *Paraburkholderia* drives phenolic acid degradation to accelerate SOC mineralization (Wilhelm et al. 2021), likely contributing to reduced SOC versus CK. LZBR primarily enriched *Bosea* (Yan et al. 2022) and unclassified *Methyloligellaceae* (Gao et al. 2025; Liu et al. 2023), which harbor plant-growth-promoting traits and resist allelochemicals via enhanced carbon/nitrogen cycling. These patterns indicate treatment-dependent microbiome restructuring that modulates soil nutrient dynamics and plant productivity.

#### FAPROTAX functional prediction

**Fig. 4:**
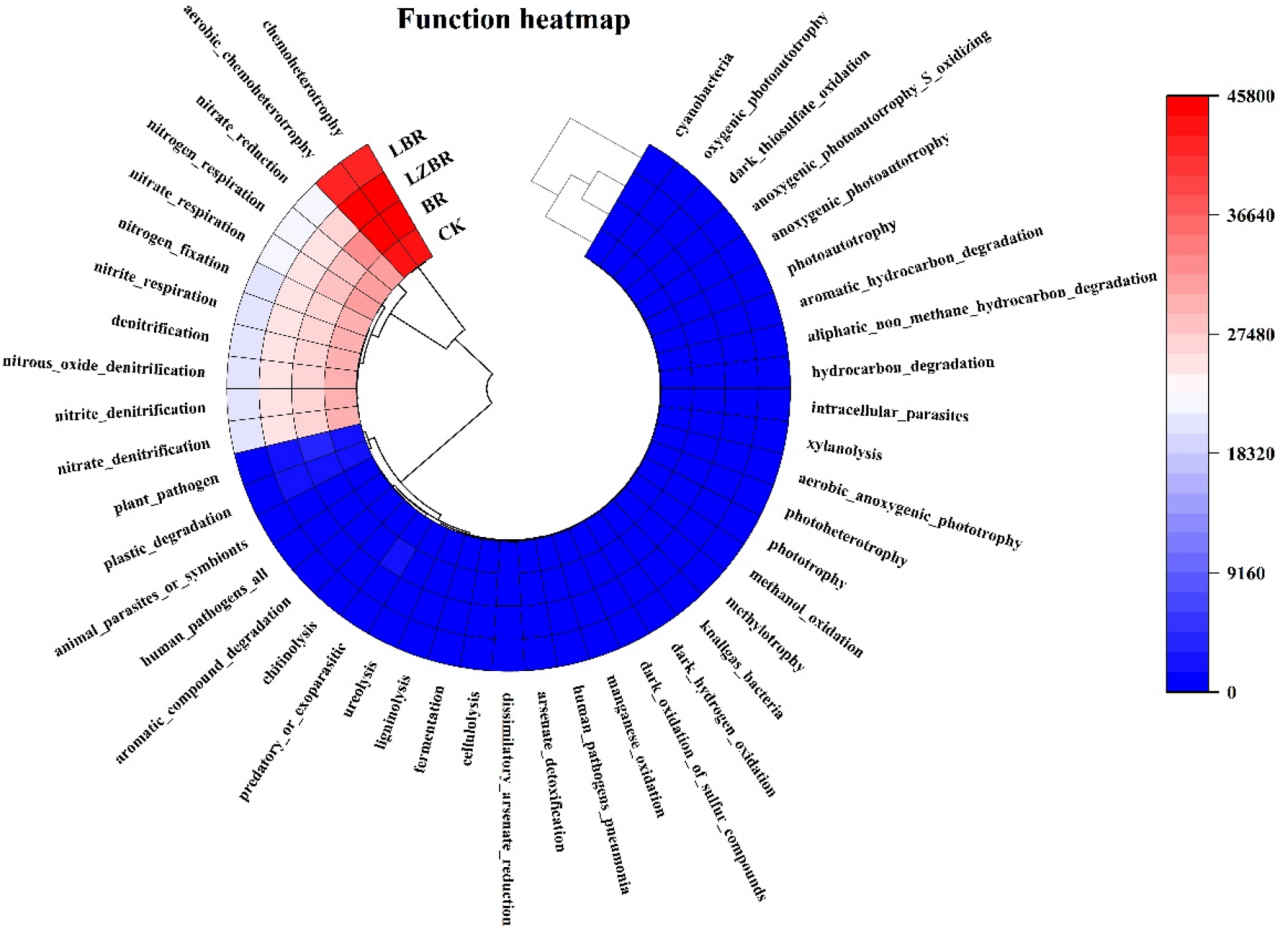
Functional gene prediction heatmap for rhizosphere microorganisms

Putative microbial functions across rhizosphere treatments were predicted using the FAPROTAX tool (Fig. 4). An elevated predicted abundance of genes related to carbon cycling (e.g., cellulose fermentation and decomposition) in BR, LBF, and LZBF treatments points to a potential trend of enhanced SOC generation, which may be supported by the organic inputs. Correspondingly, a reduced predicted abundance of nitrogen-cycling genes (e.g., for denitrification and nitrate reduction) could imply a lower potential for nitrogen loss, offering a plausible microbial-level context for the observed higher soil TKN compared to CK. Notably, genes linked to dissimilatory arsenate reduction and detoxification were significantly enriched in LBF/LZBF. This suggests a hypothetical link whereby arsenate reduction, potentially coupled to phosphate mobilization as proposed elsewhere (Ma and Zhang 2023), might facilitate the release of adsorbed phosphorus into plant-available forms, thereby potentially contributing to the elevated soil TP. We emphasize that these are functional inferences derived from taxonomic profiles; they indicate potential metabolic capacities rather than confirmed process rates, which would require direct measurement (e.g., of enzyme activities or transcriptomics) for validation.

### Soil health risk assessment

#### Heavy metals

The ecological risks of heavy metals in rhizosphere soils were assessed using the geo-accumulation index (*I*_geo_) and the potential ecological risk index (RI). Consistently negative *I*_geo_ values (Table 3) indicate an unpolluted status (Class 0: *I*_geo_ ≤ 0). Although RI values were elevated relative to CK, all individual metal risk indices remained below 40, defining a low-risk level (Chen et al. 2019). Collectively, these results suggest a low overall contamination risk under the experimental conditions and support the role of cyanobacterial biochar, CGE, and CBGE in mitigating soil pollution.

**Table 3:** Heavy metal risk assessment metrics

Index	Group	Cd	Cr	Cu	Ni	Pb
Heavy metal accumulation index	CK	≤ 0	≤ 0	− 2.928	≤ 0	≤ 0
	BR	≤ 0	≤ 0	≤ 0	≤ 0	≤ 0
	LBR	≤ 0	≤ 0	− 3.155	≤ 0	≤ 0
	LZBR	≤ 0	≤ 0	− 0.459	− 2.624	≤ 0
Potential ecological risk index	CK	0	0	0.985	0	0
	BR	0	0	0	0	0
	LBR	0	0.053	0.842	0	0.744
	LZBR	0	0	5.456	0	0

Human exposure risks were evaluated via non-carcinogenic hazard indices (HI) and carcinogenic risk coefficients (CR). As summarized in Table S4, HI values for all five metals were substantially below the safety threshold of 1.0. Treatments LBR and LZBR, in particular, reduced the copper-specific hazard compared to CK. For carcinogenic risk, only LBR exhibited a marginally detectable chromium risk (2.7 × 10^−6^; 95% CI 1.8–3.6 × 10^−6^), which remains orders of magnitude below the regulatory threshold of 1 × 10^−4^. No carcinogenic risks were detected in other treatments. Therefore, the application of CGE and CBGE did not increase human health risks and demonstrated potential for mitigating specific metal hazards. The assessment using the geo-accumulation index (*I*geo ≤ 0) and potential ecological risk index provides preliminary evidence that significant heavy metal accumulation and risk are unlikely under the current application regime.

#### Polycyclic aromatic hydrocarbons

**Fig. 5:**
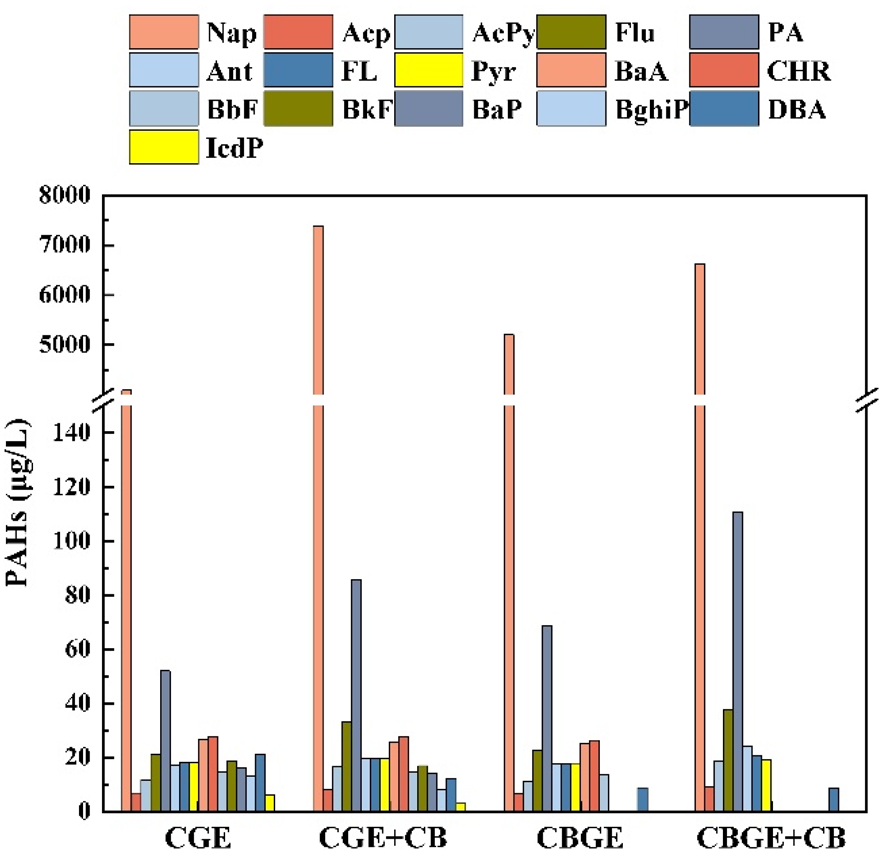
PAHs changes before (CGE: raw solution; CBGE: raw solution) and after (+ CB: post-adsorption supernatant) adsorption on biochar

This study assessed the potential environmental release of polycyclic aromatic hydrocarbons (PAHs) from cyanobacterial biochar during the preparation of CGE and CBGE composites by quantifying residual PAHs in solution before and after adsorption. Following adsorption, the total PAH concentration increased by 3334 µg/L in the CGE solution and by 1437 µg/L in the CBGE solution (Fig. 5), indicating leaching from the biochar matrix. This release is consistent with established mechanisms whereby PAH formation during pyrolysis can facilitate their mobilization into the aqueous phase (Chen et al. 2019). Notably, concentrations of naphthalene and acenaphthylene increased substantially. In contrast, levels of benzo[a]anthracene, chrysene, and benzo[b]fluoranthene in the post-adsorption CBGE solution fell below the detection limit, suggesting their selective adsorption by the biochar; given their initially low concentrations, this sequestration effect was negligible.

The observed release confirms that cyanobacterial biochar can be a source of PAHs, underscoring the necessity of evaluating its release potential prior to any field application. Regarding long-term environmental risk, translating this release potential into actual soil accumulation depends on the complex interplay between input, sequestration, and degradation dynamics. Therefore, a definitive assessment of cumulative risk under repeated applications requires future direct monitoring of PAH fate in soil–plant systems.

### Changes in crop quality

#### Soybean seed protein content

**Fig. 6:**
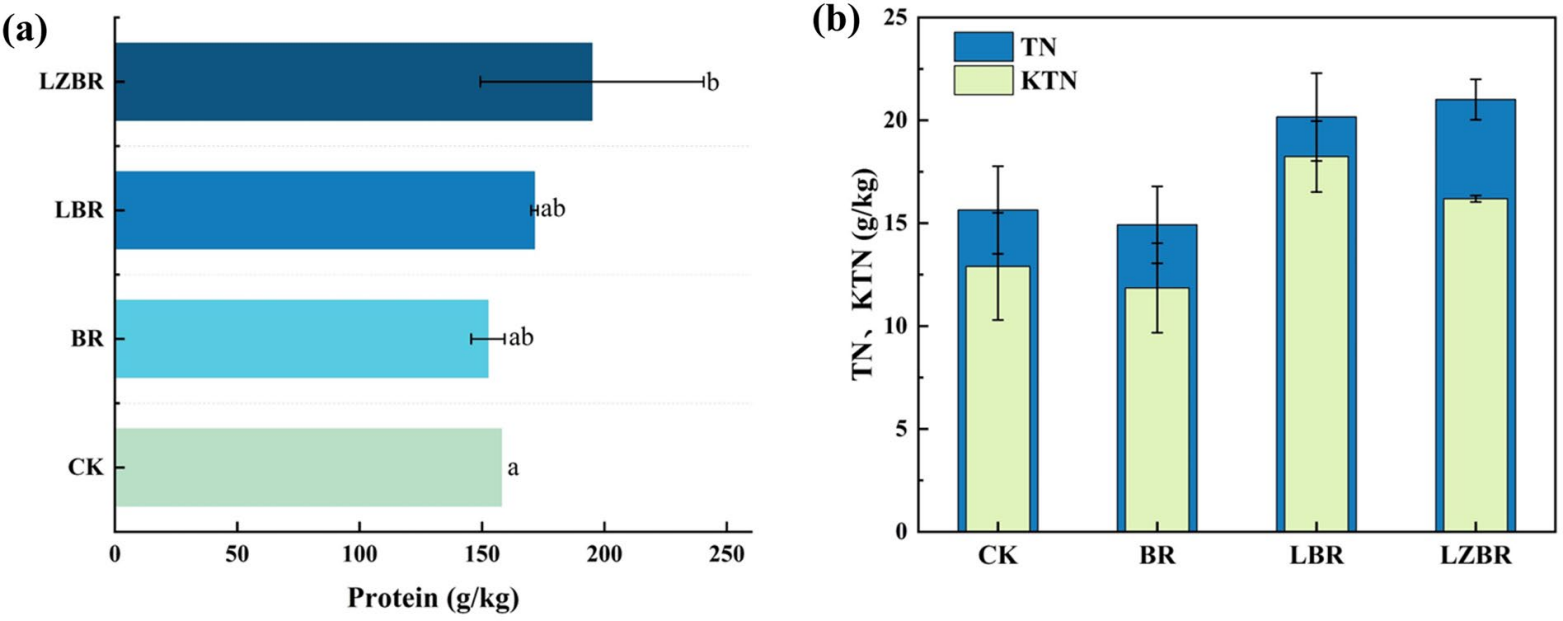
Crop quality modifications. **a** Protein content; **b** Plant nitrogen accumulation

Figure 6a shows the effects of fertilizer regimens on soybean protein content. Relative to CK, BR exhibited reduced protein content, likely due to altered soil nutrient dynamics limiting plant development. In contrast, LBR and LZBR demonstrated elevated protein concentrations of 171.4 ± 1.3 g/kg and 195 ± 46 g/kg, respectively. The relatively high standard deviation observed for the LZBR treatment may be attributed to pot-to-pot microenvironmental heterogeneity and the challenge of achieving perfect homogeneity in the distribution of the biochar-extract complex within the soil, factors inherent to pot-scale experiments. Thus, these results are best interpreted as indicative of a general trend. This increase primarily correlates with nitrogenous compounds in CGE and CBGE, which undergo gradual mineralization, providing sustained nitrogen flux during grain filling. Furthermore, the observed increase in protein content coincided with improved soil fertility metrics under LBR and LZBR treatments, which may have promoted plant growth and protein biosynthesis. Notably, LZBR achieved the highest protein content, reflecting the high organic matter content in CBGE-derived bamboo powder and its role in enhancing soil phosphorus/potassium bioavailability, thereby improving plant nutrient acquisition efficiency.

#### Plant nitrogen accumulation

Figure 6b shows the effects of different treatments on plant TKN and TN content. Relative to CK, the BR treatment showed significant reductions in plant TKN (12 ± 2 g/kg) and TN (15 ± 3 g/kg). These reductions, consistent with the lower protein content, indicate that the addition of cyanobacterial biochar alone (BR treatment) reduced plant nitrogen acquisition efficiency even in the context of elevated soil TKN. Conversely, LBR and LZBR exhibited substantial increases: TKN and TN reached 18 ± 2 g/kg and 20 ± 2 g/kg in LBR, and 16.2 ± 0.2 g/kg and 21.0 ± 0.2 g/kg in LZBR. This enhancement is primarily attributed to the nitrogenous compounds in CGE and CBGE, which increase soil nitrogen availability and provide a sustained nitrogen flux during critical growth stages. Furthermore, these CGE/CBGE amendments promote the conversion of soil TKN to plant-available forms. Although the resultant soil TKN accumulation under LBR/LZBR remained lower than in BR, these treatments improved overall nitrogen assimilation efficiency by enhancing both uptake and utilization pathways.

## Conclusions

The valorization of agricultural and forestry residues demonstrates significant potential for global carbon mitigation. CGE and CBGE derived from acid-hydrolyzed cyanobacterial biomass and bamboo powder contain substantial plant-available nutrients. The soil amendments composed of CGE or CBGE together with cyanobacterial biochar improved soil physicochemical properties, fertility, and carbon sequestration capacity in rhizosphere environments. Microbiome analyses revealed *Chryseobacterium* enrichment—a genus mediating organic matter decomposition—which, combined with taxonomic divergence and functional gene predictions, points to stimulated carbon‑nitrogen‑phosphorus cycling as a central mechanism driving soil improvement. Risk assessments confirmed negligible ecosystemic and human health risks, suggesting net environmental safety. Crucially, CBGE consistently outperformed CGE, with both treatments exceeding CK and BR outcomes. This validates a novel waste‑to‑value strategy that simultaneously addresses soil health and sustainable intensification, offering a scalable model for low‑carbon agriculture.

## Limitations and future perspectives

The findings demonstrate the efficacy of cyanobacterial‑ and bamboo‑derived amendments in a pot system. The limited number of replicates, while sufficient to detect major treatment effects, reduces statistical power for resolving more subtle differences in soil nutrient parameters. Furthermore, the single-season duration precludes assessment of long-term outcomes, including cumulative benefits or risks from repeated application. Consequently, future research will involve multi‑season field trials with robust replication to validate agronomic benefits and monitor long‑term environmental impacts. Integrated multi‑omics analyses will be implemented to define the precise functional pathways underlying the observed effects. This trajectory is directed toward transforming the approach into a proven, safe, and practical field technology.

## Supplementary Information

Additional file1 (DOCX 122 KB)

## Data Availability

The datasets generated during and/or analyzed during the current study are available from the corresponding author on reasonable request.
