# Supplementary material for: Coupled rhizosphere application of cyanobacteria-bamboo acid hydrolysis extract and cyanobacterial biochar enhances soil health and crop quality
Source: Bioresour Bioprocess. 2026 Feb 11;13(1):22. doi: 10.1186/s40643-026-01016-5 (PMC12891320; doi:10.1186/s40643-026-01016-5)
Supplement: Supplementary file 1 — Additional file1 (DOCX 122 KB) [file 40643_2026_1016_MOESM1_ESM.docx]

*Supplementary Materials*

**Coupled rhizosphere application of cyanobacteria-bamboo acid hydrolysis extract and cyanobacterial biochar enhances soil health and crop quality**

Huichang Bian ^1^, Yuzhi Li ^1^, Yibiao Zhang ^1^, Yao Shen ^1^, Jiahou Hao ^1,2^, Shuo Wang ^1,3^*, Ji Li ^1,3^

1 Jiangsu Key Laboratory of Anaerobic Biotechnology, School of Environment and Ecology, Jiangnan University, Wuxi, 214122, China

2 Department of Civil Engineering and Architecture, Nanyang Normal University, Nanyang 473061, China

3 Jiangsu College of Water Treatment Technology and Material Collaborative Innovation Center, Suzhou, 215009, China

^*^Corresponding author: E-mail: shuowang@jiangnan.edu.cn

**This file includes:**

Table S1. Background concentrations of soil heavy metals.

Table S2. BET textural properties of cyanobacterial biochar post-adsorption.

Table S3. Alpha-diversity indices of microbial communities.

Table S4. Human health risk quantification results for heavy metals.

Text S1. Supplementary materials and methods.

1. Synthesis of Cyanobacterial Growth Elicitor (CGE) and Cyanobacteria-Bamboo Growth Elicitor (CBGE).
2. Quantification methodologies for geo-accumulation index (I_geo_), potential ecological risk index (PI), and health risk assessment.

Figure S1. Rhizosphere microbial community composition at phylum taxonomic level.

Table S1. Background concentrations of soil heavy metals.

| Heavy metal types | Cd | Cr | Ni | Pb | Cu |
| --- | --- | --- | --- | --- | --- |
| Concentration（mg/kg） | 0.11 | 20.6 | 9.3 | 25.9 | 12.7 |

Table S2. BET textural properties of cyanobacterial biochar post-adsorption.

|  | Average pore size | Specific surface area | Total pore volume |
| --- | --- | --- | --- |
| Before adsorption of cyanobacterial biochar | 13.479 | 11.195 | 0.033 |
| After adsorption of CGE | 11.801 | 15.306 | 0.037 |
| After adsorption of CBGE | 7.820 | 15.035 | 0.026 |

Table S3. Alpha-diversity indices of microbial communities.

|  | ace | chao | coverage | shannon | simpson |
| --- | --- | --- | --- | --- | --- |
| CK | 274.0215 | 273.0429 | 0.999358 | 2.785241 | 0.142376 |
| BR | 240.979 | 238.2708 | 0.999485 | 2.507932 | 0.280269 |
| LBR | 355.8894 | 361.0346 | 0.999069 | 3.224083 | 0.124654 |
| LZBR | 268.0432 | 274.7899 | 0.999492 | 2.648683 | 0.245495 |

Table S4. Human health risk quantification results for heavy metals.

| Hazard(HI) | | Cu | NI | Cr | Hg | Pb |
| --- | --- | --- | --- | --- | --- | --- |
| CK | Children | 6.35×10^-5^ | 0 | 0 | 0 | 0 |
|  | Adult | 3.72×10^-5^ | 0 | 0 | 0 | 0 |
| BR | Children | 0 | 0 | 0 | 0 | 0 |
|  | Adult | 0 | 0 | 0 | 0 | 0 |
| LBR | Children | 5.43×10^-5^ | 0 | 0 | 0 | 0 |
|  | Adult | 3.18×10^-5^ | 0 | 0 | 0 | 0 |
| LZBR | Children | 3.51×10^-4^ | 1.14×10^-4^ | 0 | 0 | 0 |
|  | Adult | 2.06×10^-4^ | 6.73×10^-5^ | 0 | 0 | 0 |
| Carcinogenic risk index (CR) | | Cr | | | | |
| LBR | Children | 2.29×10^-6^ | | | | |
|  | Adult | 1.34×10^-6^ | | | | |

Text S1. Supplementary materials and methods.

1. Synthesis of Cyanobacterial Growth Elicitor (CGE) and Cyanobacteria-Bamboo Growth Elicitor (CBGE).

The cyanobacterial biomass was obtained from a dewatering operation in Wuxi, with moisture content adjusted to 98%. Acid hydrolysis was subsequently conducted in a glass reactor equipped with a thermoelectric jacket. A 2% oxalic acid solution (calculated based on cyanobacterial dry mass) and sulfuric acid were employed to adjust the pH to 0.8. The reaction proceeded at 100 °C with continuous agitation (90-100 rpm) for 24 h. Following solid residue removal and fresh biomass introduction, three to four cycles were performed to concentrate nutrients and biostimulants in the cyanobacterial growth elicitor (CGE), with the resultant supernatant designated as CGE.

Building upon the CGE synthesis protocol, cyanobacterial-bamboo growth elicitor (CBGE) was prepared by supplementing bamboo powder at a 1:1 dry mass ratio (bamboo:cyanobacteria). Semi-continuous hydrolysis utilized 2% oxalic acid, with sulfuric acid maintaining pH at 0.7-0.8. After 24-h hydrolysis cycles, solid residues were replaced with fresh biomass-bamboo mixtures. Through four repetitions, nutrients and bioactive compounds were concentrated in CBGE, and the final supernatant served as CBGE.

2. Quantification methodologies for geo-accumulation index (I_geo_), potential ecological risk index (PI), and health risk assessment.

1）I_geo_ quantification methodology is defined as follows.

I_geo_ for soil heavy metals was originally developed by Müller et al.[1]. This methodology has subsequently been extensively utilized in heavy metal contamination assessment within soil systems. The computational formula is expressed as follows:

*I_geo_*=log_2_[*C_i_*/(k×*B_i_*)]

Where C_i_ denotes the measured content of element *i* (10^-6^), B_i_ represents the regional background value of element *i* (10^-6^) as detailed in Table S1, and *k* is the lithological correction factor conventionally assigned a value of 1.5.

I_geo_ classifies pollution levels as follows: I_geo_ ≤ 0 (unpolluted); 0 < I_geo_ ≤ 1 (low-moderate pollution); 1 < I_geo_ ≤ 2 (moderate pollution); 2 < I_geo_ ≤ 3 (moderate-heavy pollution); 3 < I_geo_ ≤ 4 (heavy pollution); 4 < I_geo_ ≤ 5 (heavy-extreme pollution); I_geo_ > 5 (extreme pollution) [2].

2）The quantification methodology for the PI is defined as follows

Håkanson [3] developed the PI, a widely adopted methodology for assessing ecological risks of metals in environmental systems. The formulation is expressed as:

$$PI=\sum E_{i}=\sum T_{r, i}\times C_{f, i}=\sum T_{r,i}\times\frac{C_{i}}{C_{n,i}}$$

In the computational framework, PI quantifies the comprehensive potential ecological risk index; E_i_ defines the potential ecological risk index for heavy metal *i*; T_r,i_ denotes the toxicity response coefficient for heavy metal *i*, reflecting inherent toxicological properties and ecological sensitivity (standardized values: Cd=30, Cr=2, Ni=5, Pb=5, Cu=5); C_f,i_ represents the contamination factor for heavy metal *i*, where C_i_ indicates the measured concentration and C_n,i_ signifies the reference benchmark (typically regional background values, detailed in Table S1). Individual metal ecological risk tiers classify according to E_i_ thresholds: E_i_ < 40 (low risk); 40 ≤ E_i_ < 80 (moderate risk); 80 ≤ E_i_ < 160 (substantial risk); 160 ≤ E_i_ < 320 (high risk); E_i_ ≥ 320 (extreme risk). The composite PI characterizes multi-metal contamination risks through four classification levels: PI < 150 (low risk); 150 ≤ PI < 300 (moderate risk); 300 ≤ PI < 600 (high risk); PI ≥ 600 (very high risk) [4].

3）The health risk assessment methodology was established as follows

Human health risk assessment quantifies the probability of adverse health effects in populations (children and adults) exposed to heavy metal contaminants. Following the United States Environmental Protection Agency (USEPA) methodology for soil exposure risk assessment, primary heavy metal exposure pathways include: hand-to-mouth ingestion, inhalation of particulate matter, dermal absorption. The average daily intake (ADI) for each exposure route is calculated using standard toxicological models:

AD_i_= (C×R_i_×EF×ED) ×10^-6^/(BW×AT)

AD_b_= (C×Rb×EF×ED) ×10^-6^/(PEF×BW×AT)

AD_s_= (C×SA×SL×ABF×EF×ED) ×10^-6/^(BW×AT)

In the formulas, AD_i_, AD_b_, and AD_s_ represent the average daily exposure doses via hand-to-mouth ingestion, inhalation, and skin contact, respectively (mg/(kg·d)). C denotes the heavy metal content in soil. Parameter definitions and reference values are documented in Table S2.

Soil heavy metals primarily induce carcinogenic and non-carcinogenic risks to humans, characterized by the carcinogenic risk index (CR) and non-carcinogenic hazard index (HI), respectively [4]. The calculation formulas are defined as follows:

*HQ_i_=ADD_i_*/*RfD_i_*

*HI=∑HQ_i_*

*CR=∑ADD_i_×SF_i_*

In the computational framework, HQ_i_ defines the non-carcinogenic hazard index for individual heavy metals; ADD_i_ quantifies the average daily exposure dose for non-carcinogenic metals; RfD_i_ indicates the reference dose; SF_i_ signifies the carcinogenic slope factor. Parameter reference values are cataloged in Table S3. For non-carcinogenic risk assessment, HQ or HI values exceeding 1 denote negligible risk, while values ≤1 indicate potential non-carcinogenic hazards. Regarding carcinogenic risks, CR values below 10⁻⁶ represent negligible risk; values between 10⁻⁶ and 10⁻⁴ correspond to low-risk conditions; values surpassing 10⁻⁴ signify high-risk scenarios [4, 5].

Table S5. Health risk assessment exposure parameters.

| Parameter definitions | Unit | Children | Adults |
| --- | --- | --- | --- |
| Daily intake rate (*R_i_*) | mg/d | 200 | 100 |
| Exposure frequency (*EF*) | d/a | 350 | 350 |
| Exposure duration (*ED*) | a | 6 | 25 |
| Average body weight (*BW*) | kg | 15.9 | 56.8 |
| Averaging time (*AT*) | d/a | 26280 | 26280 |
| Daily inhalation rate (*Rb*) | m^3^/d | 7.5 | 14.5 |
| Particulate emission factor (*PEF*) | m^3^·kg | 1.36×10^9^ | 1.36×10^9^ |
| Skin surface area exposed (*SA*) | cm^2^ | 2800 | 5700 |
| Soil adherence factor (*SL*) | mg/cm^2^ | 0.2 | 0.07 |
| Soil adherence factor (*ABL*) | Dimensionles | 0.001 | 0.001 |

Table S6. Reference doses and carcinogenic slope factors for metal elements.

| Heavy metal types | *RfD_i_* | *SF_i_* |
| --- | --- | --- |
| Cr | 2.86×10-5 | 4.2 |
| Cd | 2.55×10-5 | - |
| Pb | 3.52×10-4 | - |

References

[1] Muller, G. M. M. G. M. G. M. G. P. (1969). Index of geoaccumulation in sediments of the Rhine River.

[2] Charzyński, P., Plak, A., & Hanaka, A. (2017). Influence of the soil sealing on the geoaccumulation index of heavy metals and various pollution factors. Environmental Science and Pollution Research, 24, 4801-4811.

[3] Hakanson, L. (1980). An ecological risk index for aquatic pollution control. A sedimentological approach. Water research, 14(8), 975-1001.

[4] Cui, Y., Bai, L., Li, C., He, Z., & Liu, X. (2022). Assessment of heavy metal contamination levels and health risks in environmental media in the northeast region. Sustainable Cities and Society, 80, 103796.

[5] Liang, N., Zhang, C., Zhang, Y., Jiang, N., Yang, J., Geng, N., ... & Chen, J. (2025). Pollution levels, distribution characteristics and risk assessment of heavy metals (HMs) in road dust in major cities of China. Journal of Hazardous Materials, 138733.


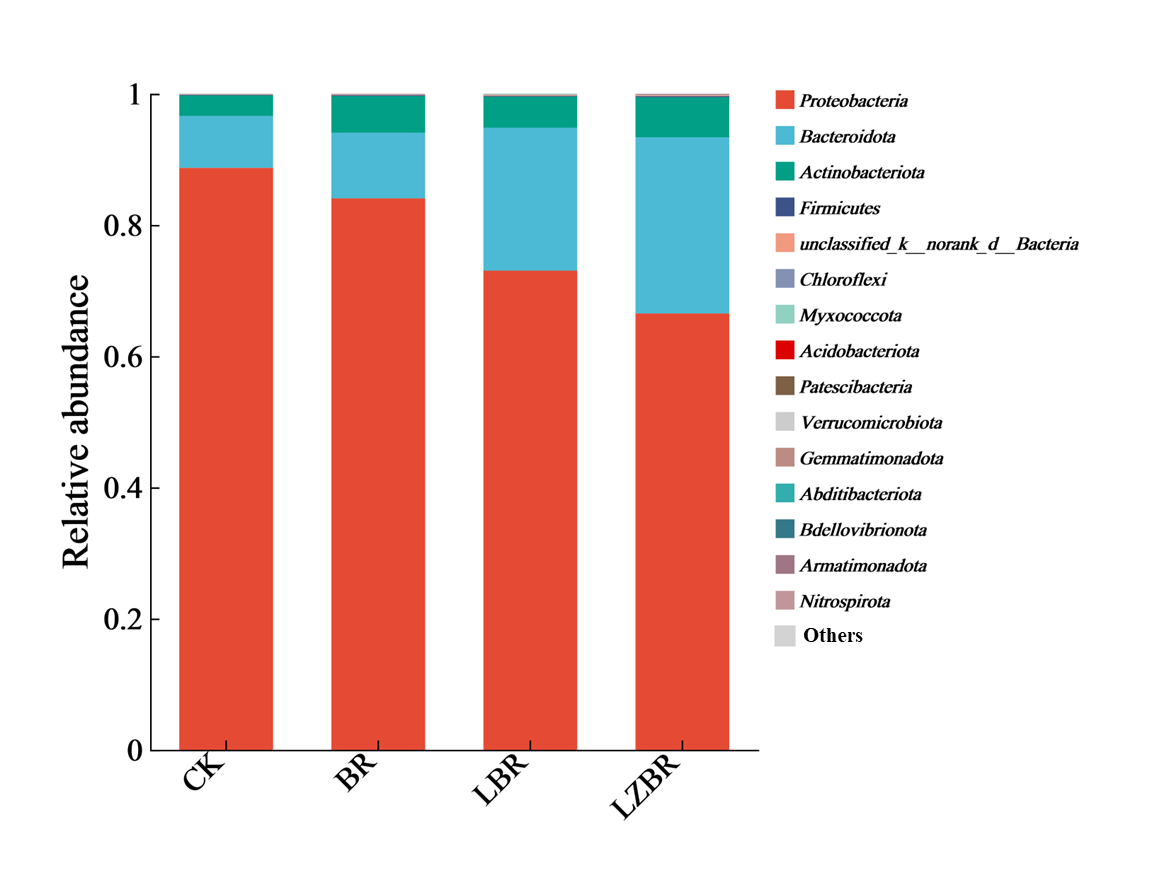


Figure S1. Rhizosphere microbial community composition at phylum taxonomic level.
